# ESX Secretion-Associated Protein C From *Mycobacterium tuberculosis* Induces Macrophage Activation Through the Toll-Like Receptor-4/Mitogen-Activated Protein Kinase Signaling Pathway

**DOI:** 10.3389/fcimb.2019.00158

**Published:** 2019-05-10

**Authors:** Qinglong Guo, Jing Bi, Ming Li, Wenxue Ge, Ying Xu, Weixing Fan, Honghai Wang, Xuelian Zhang

**Affiliations:** ^1^State Key Laboratory of Genetic Engineering, School of Life Science, Fudan University, Shanghai, China; ^2^Key Laboratory of Medical Molecular Virology, Ministry of Education and Health, School of Basic Medical Sciences, Fudan University, Shanghai, China; ^3^Laboratory of Zoonosis, China Animal Health and Epidemiology Center, Qingdao, China

**Keywords:** *Mycobacterium tuberculosis*, ESX secretion-associated protein C, macrophage activation, Toll-like receptor 4, mitogen-activated protein kinase

## Abstract

*Mycobacterium tuberculosis*, as a facultative intracellular pathogen, can interact with host macrophages and modulate macrophage function to influence innate and adaptive immunity. Proteins secreted by the ESX-1 secretion system are involved in this relationship. Although the importance of ESX-1 in host-pathogen interactions and virulence is well-known, the primary role is ascribed to EsxA (EAST-6) in mycobacterial pathogenesis and the functions of individual components in the interactions between pathogens and macrophages are still unclear. Here, we investigated the effects of EspC on macrophage activation. The EspC protein is encoded by an *espA/C/D* cluster, which is not linked to the *esx-1* locus, but is essential for the secretion of the major virulence factors of ESX-1, EsxA and EsxB. Our results showed that both EspC protein and EspC overexpression in *M. smegmatis* induced pro-inflammatory cytokines and enhanced surface marker expression. This mechanism was dependent on Toll-like receptor 4 (TLR4), as demonstrated using EspC-treated macrophages from TLR4^−/−^ mice, leading to decreased pro-inflammatory cytokine secretion and surface marker expression compared with those from wild-type mice. Immunoprecipitation and immunofluorescence assays showed that EspC interacted with TLR4 directly. Moreover, EspC could activate macrophages and promote antigen presentation by inducing mitogen-activated protein kinase (MAPK) phosphorylation and nuclear factor-κB activation. The EspC-induced cytokine expression, surface marker upregulation, and MAPK signaling activation were inhibited when macrophages were blocked with anti-TLR4 antibodies or pretreated with MAPK inhibitors. Furthermore, our results showed that EspC overexpression enhanced the survival of *M. smegmatis* within macrophages and under stress conditions. Taken together, our results indicated that EspC may be another ESX-1 virulence factor that not only modulates the host innate immune response by activating macrophages through TLR4-dependent MAPK signaling but also plays an important role in the survival of pathogenic mycobacteria in host cells.

## Introduction

Tuberculosis, caused by *Mycobacterium tuberculosis*, is related to high morbidity and mortality rates and is a critical health concern worldwide (World Health Organization, 2016). Once infected with *M. tuberculosis*, hosts will initiate innate and adaptive immune responses against mycobacteria to restrict bacterial survival and eliminate the bacterium (Akira et al., 2001). However, *M. tuberculosis* can escape host immune defense and replicate within permissive macrophages through multiple strategies, including prevention of phagolysosome maturation, tolerance to the acidic environment of phagolysosomes, and inhibition of apoptosis and autophagy (Lee et al., 2009; Levitte et al., 2016; Saini et al., 2016). Importantly, *esx-1* genes, which encode either ESX core complexes or ESX secretion-associated proteins (Esps), form a novel bacterial VII secretion system (T7S) involved in virulence factor export and host-pathogen interactions. Region of difference 1 (RD1) is a part of the larger *esx-1* locus and is present in virulent *M. tuberculosis* and *M. bovis* but absent from all *M. bovis* BCG vaccine strains. Deletion of RD1 causes attenuated virulence in macrophages and experimental animals. EsxA (also named 6-kDa-early-secreted antigenic target [ESAT-6]) is a well-known virulence factor of ESX-1 or RD1 in pathogenic mycobacteria and participates in host-pathogen interactions (Van Pinxteren et al., 2000; Brodin et al., 2005). The functional roles of EsxA have been linked to membrane lysis, enabling the phagosomal escape of bacteria. However, research on whether there are other ESX-1 secreted proteins that also play an important role in the interaction between the host and mycobacteria is limited.

EsxA/B secretion depends on the presence of several ESX-1 substrate proteins (Esps) (Fortune et al., 2005; McLaughlin et al., 2007; Raghavan et al., 2008). EspA, EspC, and EspD, which form a genetic cluster that is located more than 260 kb upstream of the *esx-1* locus, are also ESX-1 substrates and are essential for EsxA/B secretion (Fortune et al., 2005; Raghavan et al., 2008). In fact, EspA/C secretion also depends on EsxA/B, and EspA/C and EsxA/B are secreted in a mutually dependent manner (Fortune et al., 2005; Millington et al., 2011). Moreover, the *espACD* locus is highly conserved and restricted to pathogenic mycobacteria, including *M. leprae*, which has a relatively smaller genome. EspC (Rv3615c) has a size and sequence homology similar to those of EsxA and EsxB and has a YxxxD motif, which is necessary for secretion of proteins in T7S (Son et al., 2016). A recent study showed that EspC forms a bacterial surface-exposed filamentous structure in *M. tuberculosis* that may act as an ESX-1 secretion channel for secretion of proteins, such as EsxA/B (Lou et al., 2017). Thus, we proposed that EspC may also be an important factor contributing to the virulence of pathogenic mycobacteria.

Similar to ESAT-6, EspC is a highly specific T-cell antigen, which is a potential tuberculosis vaccine candidate and may be applied in T-cell-based immunodiagnosis in BCG-vaccinated populations and cattle (Sidders et al., 2008; Millington et al., 2011). Millington et al. and others have shown that EspC can be a potent differential diagnostic antigen in both active and latent TB infections, and T-cell responses to EspC are highly specific (93%) for *M. tuberculosis* infection (Millington et al., 2011). Cocktails of ESAT-6/CFP-10 and EspC are used not only for differential diagnosis of para-tuberculosis mycobacteria or BCG vaccination and *M. bovis* infection in cattle, but also to distinguish infected cattle from non-tuberculosis mycobacteria-exposed uninfected animals, suggesting that EspC could be an essential antigen for the diagnosis of bovine tuberculosis (Sidders et al., 2008; Serrano et al., 2017; Jenkins et al., 2018). The high immunodominance of EspC, equivalent to that of ESAT6 and CFP10, and its high antigenic specificity raise a possibility that EspC could also play critical roles in EspC and macrophage interaction during pathogenic mycobacterial infections. However, the interactions between EspC and macrophages are still poorly understood. We hypothesize that EspC may directly interact with pattern recognition receptors on macrophages, participate in host immunoregulation, and contribute to the early spread of mycobacteria.

The aim of the present study was to characterize the biological effects of EspC on macrophages. We confirmed that EspC was another factor from the ESX-1 system, which interacted directly with TLR4 and greatly affected macrophage activation and antigen presentation via MAPK pathways to activate the innate immune response and EspC could improve the survival of *M. smegmatis* within macrophages.

## Materials and Methods

### Mice and Cell Lines

C57BL/6 mice were purchased from the Animal Center of Slaccas (Shanghai, China). TLR4^−/−^ mice (6–8 weeks old) were obtained from the Model Animal Research of Nanjing University (Nanjing, China). All mice were maintained under specific pathogen-free conditions in the Animal Center of the School of Life Science of Fudan University and used according to the Guide for the Care and Use of Laboratory Animals of the National Institutes of Health. The experimental protocol was approved by the Animal Care and Use Committee of Fudan University. The RAW264.7 cell line was originally purchased from the American Type Culture Collection (Manassas, VA, USA). RAW264.7 cells were cultured in Dulbecco's modified Eagle's medium (DMEM; Gibco, Grand Island, NY, USA) supplemented with 10% fetal bovine serum (FBS), penicillin (100 U/mL), and streptomycin (100 mg/mL) and maintained at 37°C in a humidified incubator (5% CO_2_). THP-1 cells were cultured in Roswell Park Memorial Institute-1640 (Gibco) supplemented with 10% FBS, penicillin (100 U/mL), and streptomycin (100 mg/mL) and maintained at 37°C in a humidified incubator (5% CO_2_). THP-1 cells (1 × 10^6^ cells/well) were seeded in 12-well tissue culture plates, and 50 ng/mL phorbol 12-myristate 13-acetate (PMA) was added to each well. After induction for 48 h, THP-1 cells were differentiated from monocytes to macrophages for specific experiments.

### Cloning, Expression, and Purification of Recombinant EspC

The gene coding *M. tuberculosis* EspC was amplified using the primer sequences (sense) 5′-GCGGATCCATGACGGAAAACTTGACCGTCCA-3′ and (antisense) 5′-CCCAAGCTTTCAGGTAAACAACCCGTCGATAGCCTT-3′. The *espC* product was digested with the restriction enzymes *BamHI* and *HindIII*, inserted into the pET28a (+) plasmid (Novagen, Madison, WI, USA), and transformed into *Escherichia coli* BL21 cells. Recombinant EspC was prepared by induction of bacterial cells with 0.5 mM isopropyl β-d-1-thiogalactopyranoside at 37°C for 12 h. The harvested cells were lysed as previously described. Recombinant EspC was purified using HIS-Select Nickel Affinity Gel (Sigma-Aldrich, St. Louis, MO, USA), following the manufacturer's instructions. To remove endotoxins, the dialyzed recombinant EspC was incubated with polymyxin B-agarose (Sigma) overnight at 4°C. The preparation had a very low endotoxin content (< 0.05 EU/mg), as measured using an E-toxate (Limulus amebocyte lysate) kit (Sigma-Aldrich). The protein concentration was estimated with a bicinchoninic acid (BCA) protein assay kit (Pierce, Rockford, IL, USA), and protein was then stored at −80°C until use.

### Measurement of Cytokine Levels by Enzyme-Linked Immunosorbent Assays (ELISAs)

Tumor necrosis factor (TNF)-α, interleukin (IL)-6, and monocyte chemoattractant protein (MCP)-1 secreted from macrophages in culture supernatants stimulated with various concentrations of EspC were measured by sandwich ELISAs (Biolegend, San Diego, CA, USA) according to the manufacturer's instructions. The cytokine levels were estimated by measuring absorbance at 450 nm with a microplate reader. Purified EspC protein (5 μg/mL) or LPS (1 μg/mL) were treated with proteinase K (PK, 20 mg/mL) overnight at 56°C. The treated EspC protein was subjected to western blot analysis using anti-His mouse antibodies. Untreated EspC protein was used as a positive control. RAW264.7 cells were incubated with EspC (0.25, 1, 5, or 10 μg/mL), EspC (5 μg/mL) treated with PK, PK alone, LPS (1 μg/mL), LPS (1 μg/mL) treated with PK, or Pam3CSK4 (5 μg/mL) for 24 h at 37°C. In TLR signaling blocking experiments, RAW264.7 cells were pretreated for 30 min at 37°C with mouse anti-TLR2 (30 μg/mL) or anti-TLR4 (30 μg/mL) antibodies or a mouse IgG isotype-matched control antibody (30 μg/mL). In pharmacological inhibitor experiments, RAW264.7 cells were pre-incubated for 1 h at 37°C with inhibitors for p38 MAPK (SB203580; 30 μM), c-Jun N-terminal kinase (JNK; SP600125; 25 μM), and extracellular signal-regulated kinase (ERK; U0126; 10 μM). RAW264.7 cells were then stimulated with EspC for 24 h at 37°C. Mouse anti-TLR2, anti-TLR4, and IgG isotype control antibodies were obtained from BioLegend, and inhibitors were purchased from Cell Signaling Technology (Beverly, MA, USA).

### Protein Isolation and Western Blot Analysis

RAW264.7 cells were incubated with 5 μg/mL EspC for the indicated times, harvested, and lysed with cell lysis buffer (Biyuntian, Nanjing, China) supplemented with protease inhibitor cocktail tablets (Roche Molecular Biochemicals, Indianapolis, IN, USA). After centrifugation at 15,000 × *g* and 4°C for 30 min, the protein concentrations in the supernatants were measured using BCA protein quantification assays. Cell pellets were processed using a NEPER Nuclear and Cytoplasmic Extraction kit (Pierce) according to the manufacturer's instructions. Equal amounts of protein were separated by sodium dodecyl sulfate (SDS)-polyacrylamide gel electrophoresis (PAGE) and then transferred onto polyvinyl difluoride membranes (Millipore, Bedford, MA, USA). The membranes were blocked in 5% nonfat milk for 1 h at room temperature and incubated overnight at 4°C with primary antibodies, including rabbit anti-ERK2, rabbit anti-JNK, rabbit anti-p38 MAPK, rabbit anti-phospho-ERK1/2, rabbit anti-phospho-JNK, rabbit anti-phospho-p38 MAPK, mouse anti-IκB-α, rabbit anti-nuclear factor (NF)-κB p65 (Cell Signaling Technology), mouse anti-β-actin, and mouse anti-Histone H3 (Biyuntian). After washing with phosphate-buffered saline (PBS) containing Tween20 (PBST) three times, the membranes were incubated with horseradish peroxidase (HRP)-conjugated goat anti-mouse IgG or HRP-conjugated goat anti-rabbit IgG secondary antibodies (1:10,000) for 2 h at room temperature. Specific protein bands were detected using Amersham ECL Prime Western Blotting Detection Reagent (GE Healthcare, Buckinghamshire, UK) and a ChemiScope 3400 mini Imaging System (Clinx Science Instruments, Shanghai, China).

### Flow Cytometry Analysis of Cell Surface Molecule Expression

RAW264.7 cells were treated, harvested, washed with PBS, and stained with 1 μg phycoerythrin (PE)-conjugated anti-mouse CD86 antibodies and fluorescein isothiocyanate (FITC)-conjugated anti-mouse CD80 or PE-conjugated anti-mouse CD40 and FITC-conjugated anti-mouse I-AK antibodies (Biolegend) for 30 min at room temperature. After washing and resuspending with PBS, the cells were analyzed using a FACSCalibur machine (Becton Dickinson, USA). The data were processed using Cell-Quest data analysis software.

### Immunoprecipitation

RAW264.7 cells (1 × 10^7^) were lysed with lysis buffer and centrifuged, and supernatants were then mixed and incubated with 5 μg/mL EspC protein. After preclearing with protein G agarose beads (GE Healthcare) for 1 h, the supernatants were incubated with anti-TLR2 and anti-TLR4 for 12 h at 4°C, and the bound proteins were then pulled down using protein G agarose beads for 4 h at 4°C. Alternatively, cell lysates were incubated with EspC immobilized on Ni-NTA beads for 12 h at 4°C. The beads were washed six times, resuspended in 1 × SDS-PAGE loading buffer, and boiled for 10 min. The proteins were separated by SDS-PAGE on 12% gels and probed with anti-mouse TLR2, anti-mouse TLR4, and anti-HIS antibodies, followed by incubation with HRP-conjugated goat anti-mouse IgG secondary antibodies. Target proteins were visualized using ECL reagent (GE Healthcare).

### Confocal Laser Scanning Microscopy

RAW264.7 cells or peritoneal macrophages derived from C57BL/6 or TLR4^−/−^ mice were plated overnight on 20-mm glass-bottom cell culture dishes. Cells were treated with EspC (5 μg/mL) for 30 min, fixed and permeabilized in Immunol Staining Fix Solution (Biyuntian) for 20 min, and blocked with 3% bovine serum albumin in PBS. After washing with PBS, the treated RAW264.7 cells were incubated with anti-rabbit TLR2, anti-rabbit TLR4, and anti-mouse HIS antibodies for 2 h at 37°C in Immunol Staining Primary Antibody Dilution Buffer (Biyuntian). Alternatively, the treated peritoneal macrophages derived from C57BL/6 or TLR4^−/−^ mice were incubated with anti-rat TLR4 and anti-mouse HIS antibodies under the same conditions. After washing, the cells were restained with appropriate Alexa Fluor 488- or Alexa Fluor 594-conjugated secondary antibodies for 1 h at 37°C. Cells were then stained with 0.1 μg/mL 4′,6-diamidino-2-phenylindole (DAPI) for 10 min at room temperature. The cells were analyzed by confocal microscopy using a Leica TCS SP8 laser scanning confocal microscope (Leica Microsystems, Wetzlar, Germany) with a 100 × oil immersion objective. Images were acquired and processed using LAS AF Lite software.

### Construction of *M. smegmatis*::*espC* (Ms::*espC*)

*espC* was ligated into the pPscreen (PSQ) shuttle vector using *BamHI* and *HindIII*. The PSQ plasmid containing *espC* and empty vector was electroporated into *M. smegmatis* mc^2^155. The recombinant Ms::*espC* was cultured in Middlebrook 7H9 with 10% oleic albumin dextrose catalase (OADC) containing 50 μg/mL kanamycin. The Ms::*espC* strain was confirmed by western blotting using mouse anti-HIS monoclonal antibodies. To investigate whether the Ms::*espC* strain secreted EspC, the recombinant Ms::*espC* strain and vector control strain were cultured in Sauton's medium containing 50 μg/mL kanamycin for 3 days, and both the bacteria and the cell culture supernatant were then harvested for EspC protein secretion analysis.

### Intracellular Survival Assays

RAW264.7 cells were seeded at a density of 1 × 10^6^ cells/well in 12-well tissue culture plates. The cells were infected with Ms::*espC* and Ms::PSQ at a multiplicity of infection (MOI) of 10:1 or 20:1 (bacteria-to-RAW264.7 cell ratio) at 37°C in an atmosphere containing 5% CO_2_ after cultivation overnight. Four hours after infection, cultures containing uninfected bacteria were removed and washed four times with warm PBS. DMEM supplemented with 10% FBS and 20 μg/mL gentamicin was then added for another 2 h. Fresh medium (DMEM supplemented with 10% FBS) was added after 2 h. At the indicated times after infection, macrophages were lysed using 1% Triton-X 100. The lysed macrophages were diluted and plated on 7H10 agar plates, and colony forming units were determined as a measure of the intracellular survival of recombinant *M. smegmatis*.

### Statistical Analysis

Data are represented as means ± standard errors of the means (SEMs) of triplicate experiments. All results were analyzed using one-way analysis of variance followed by Tukey's test or two-way analysis of variance followed by Bonferroni's test using GraphPad Prism 5.0. Differences with *p* ≤ 0.05 were considered significant.

## Results

### EspC Treatment Induced Pro-inflammatory Cytokines and Enhanced the Expression of Surface Markers in a TLR4-dependent Manner

**Figure 1:**
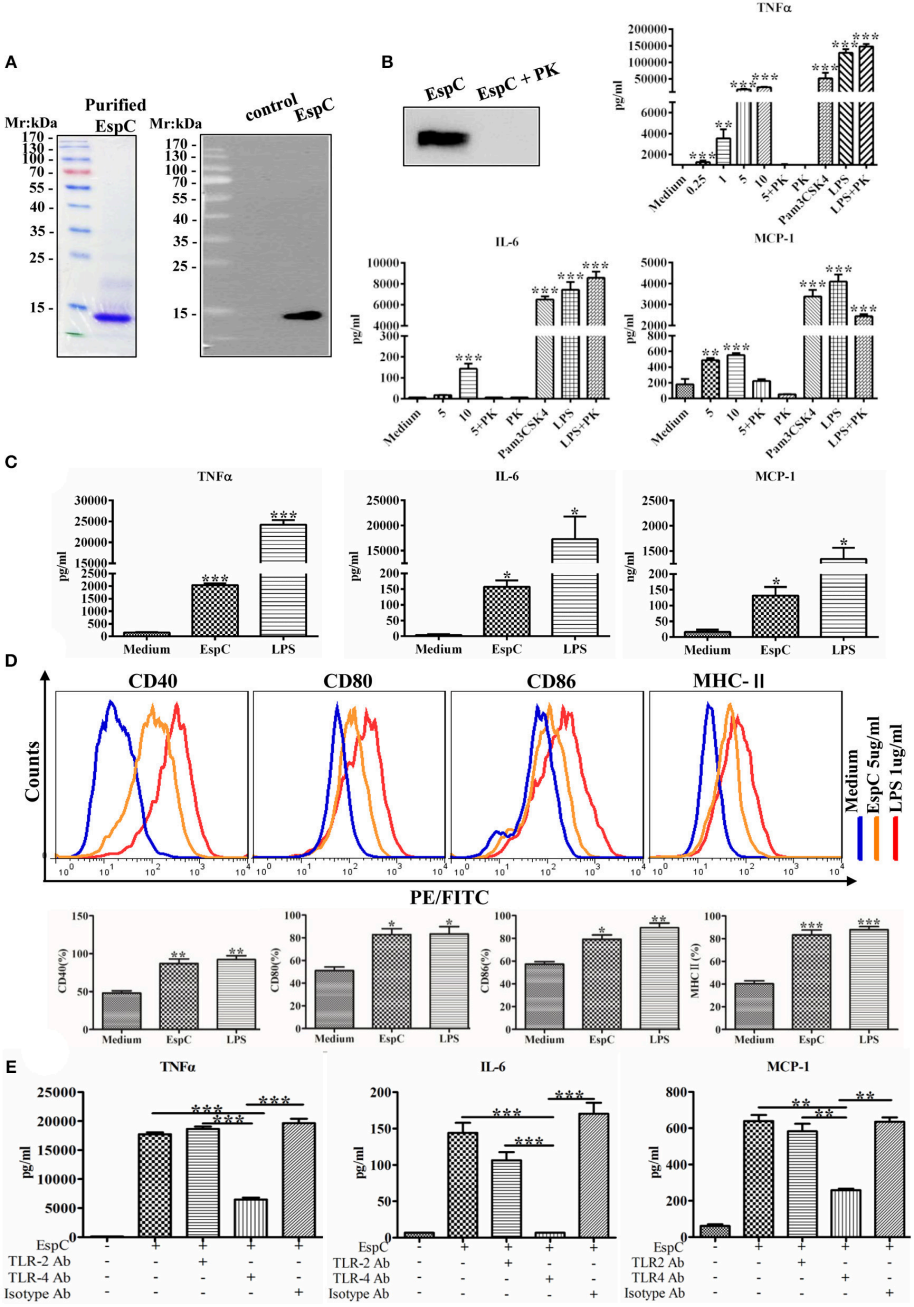
EspC induced cytokine secretion and the production of costimulatory and MHC-II molecules in macrophages. **(A)** The protein was expressed in *Escherichia coli* and purified by Ni-NTA affinity chromatography. The purified protein was subjected to SDS-PAGE analysis by staining with Coomassie blue or to western blot analysis using anti-His mouse antibodies. **(B)** Purified EspC protein (5 μg/mL) was treated with or without proteinase K overnight at 56°C and was then subjected to western blot analysis using anti-His mouse antibodies. RAW264.7 cells were incubated with EspC (0.25, 1, 5, or 10 μg/mL) and EspC (5 μg/mL) treated with proteinase K, proteinase K alone, LPS (1 μg/mL), LPS (1 μg/mL) treated with proteinase K, or Pam3CSK4 (5 μg/mL). After incubation for 24 h, cell culture supernatants were collected, and TNF-α, IL-6, and MCP-1 levels were measured by ELISA. **(C)** THP-1 cells were differentiated in the presence of PMA for 48 h and then incubated with EspC (5 μg/mL) or LPS (1 μg/mL). After incubation for 36 h, cell culture supernatants were collected, and TNF-α, IL-6, and MCP-1 levels were measured by ELISA. **(D)** The expression levels of surface markers of RAW264.7 cells, including CD80, CD86, CD40, and MHC-II, were determined at 24 h after treatment with medium alone, 5 μg/mL EspC, or LPS by FACS analysis using respective FITC- or PE-conjugated monoclonal antibodies. **(E)** RAW264.7 cells were incubated with blocking antibodies before stimulation with EspC. TNF-α, IL-6, and MCP-1 production levels were measured by ELISA. All data are expressed as means ± SEMs (*n* = 3); ^*^p < 0.05, ^**^p < 0.01, ^***^p < 0.001.

We first overexpressed and purified a His-tagged EspC in an *E. coli* expression system. The purified protein emerged as a major band at approximately 10,100 MW and reacted with the anti-His antibody on an immunoblot (Figure 1A). According to SDS-PAGE, its purity was >97% (Figure 1A).

Because EspC is a highly immunodominant RD1-dependent secreted antigen specific for mycobacteria infection, we evaluated the release of cytokines from RAW264.7 macrophages stimulated with various concentrations of EspC. As shown in Figure 1B, EspC induced significant increases in TNF-α, IL-6, and MCP-1 levels in a concentration-dependent manner. In contrast, untreated macrophages secreted insignificant levels of these cytokines. Moreover, the stimulatory effects of EspC were abolished by treating purified recombinant EspC protein with PK, and the stimulatory effects of LPS treated with PK were comparable to those of LPS alone, indicating that PK treatment did not degrade cell surface TLR4 receptor (Figure 1B). The results also suggested that EspC protein, but not LPS contamination, was responsible for the generation of pro-inflammatory cytokines. In addition, we evaluated the release of these pro-inflammatory cytokines in PMA-differentiated human monocytic THP-1 cells. The results showed that EspC also induced significant increases in TNF-α, IL-6, and MCP-1 levels in THP-1 cells (Figure 1C). These results indicated that EspC could induce macrophages to generate pro-inflammatory cytokines.

The expression of surface markers (including CD80, CD86, CD40, and MHC-II) was also measured to examine the effects of EspC on the presentation and processing of antigen. RAW264.7 cells were stimulated with EspC for 24 h and then analyzed for surface marker expression by flow cytometry. As shown in Figure 1D, the expression of CD40, CD80, CD86, and MHC-II was substantially increased after EspC stimulation compared with that in untreated macrophages, indicating that EspC induced the expression of costimulatory molecules and MHC-II. Moreover, when RAW264.7 cells were pretreated with anti-TLR4 antibodies before stimulation with EspC, the production of TNF-α, IL-6, and MCP-1 was significantly reduced. In contrast, pre-incubation with anti-TLR2 and isotype antibodies showed that there were no significant reductions in the EspC-induced production of TNF-α, IL-6, and MCP-1 (Figure 1E). These results suggested that the decreased secretion of TNF-α, IL-6, and MCP-1 in the presence of anti-TLR4 antibodies resulted from inhibition of TLR4.

**Figure 2:**
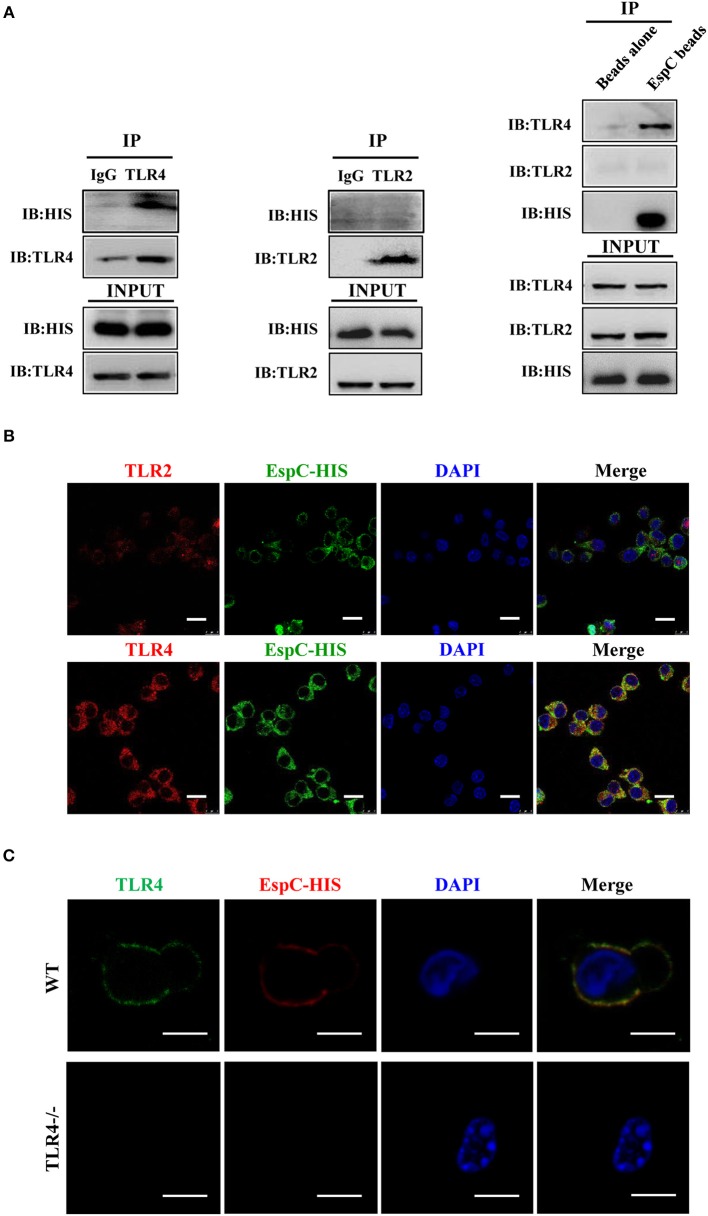
EspC bound to TLR4 but not TLR2. **(A)** Cell lysates were incubated with EspC (5 μg/mL) for 12 h and were then immunoprecipitated with anti-mouse IgG, anti-TLR2, or anti-TLR4 antibodies. The proteins were then visualized by western blotting with anti-HIS, anti-TLR2, or anti-TLR4 monoclonal antibodies. Total cell lysates were used as input control. Alternatively, cell lysates were incubated with EspC immobilized on Ni-NTA beads, and bead-bound proteins were loaded on gels for immunoblotting using anti-HIS, anti-TLR2, or anti-TLR4 monoclonal antibodies. **(B)** RAW264.7 cells were treated with EspC (5 μg/mL) for 30 min, fixed, stained with primary antibodies (mouse anti-HIS, rabbit anti-mouse TLR4, and rabbit anti-mouse TLR2 antibodies), and then re-incubated with appropriate Alexa Fluor 488- or Alexa Fluor 594-conjugated secondary antibodies and DAPI. The cells were photographed under a fluorescent microscope (original magnification: 100 × ). Scale bar = 10 μm. Data are representative of three independent experiments. **(C)** Peritoneal macrophages derived from TLR4^−/−^ mice or C57BL/6 mice were treated with EspC (5 μg/mL) for 30 min, fixed, stained with primary antibodies (mouse anti-HIS and rat anti-mouse TLR4 antibodies), and then re-incubated with appropriate Alexa Fluor 488- or Alexa Fluor 594-conjugated secondary antibodies and DAPI. The cells were photographed under a fluorescent microscope (original magnification: 100 × ). Scale bar = 5 μm. Data are representative of three independent experiments.

To further determine whether EspC interacted with TLR4 directly, we carried out immunoprecipitation assays in RAW264.7 cell lysates. Using anti-TLR4 antibodies, we found that TLR4 was coprecipitated with EspC (Figure 2A). In contrast, immunoprecipitation using anti-TLR2 antibodies showed that TLR2 was not coprecipitated with EspC (Figure 2A). Moreover, EspC-bound beads pulled down TLR4 but not TLR2 (Figure 2A). These results suggested that EspC bound to TLR4, but not TLR2. To further demonstrate that EspC bound to TLR4 specifically, RAW264.7 cells were incubated with EspC to evaluate whether EspC could bind to TLR4 using immunofluorescence microscopy. The results showed that EspC could distribute all around cells by binding to the receptor and may be colocalized with TLR4 but not with TLR2, although the labeling of TLR2 was faint compared with that of TLR4 (Figure 2B). We further used peritoneal macrophages derived from TLR4^−/−^ mice or C57BL/6 mice to evaluate whether EspC bound to TLR4 specifically. The results showed that EspC colocalized with TLR4 on macrophages derived from C57BL/6 mice, but not those from TLR4^−/−^ mice expressing TLR2 (Figure 2C). Taken together, these results indicated that EspC interacted specifically and predominantly with TLR4.

**Figure 3:**
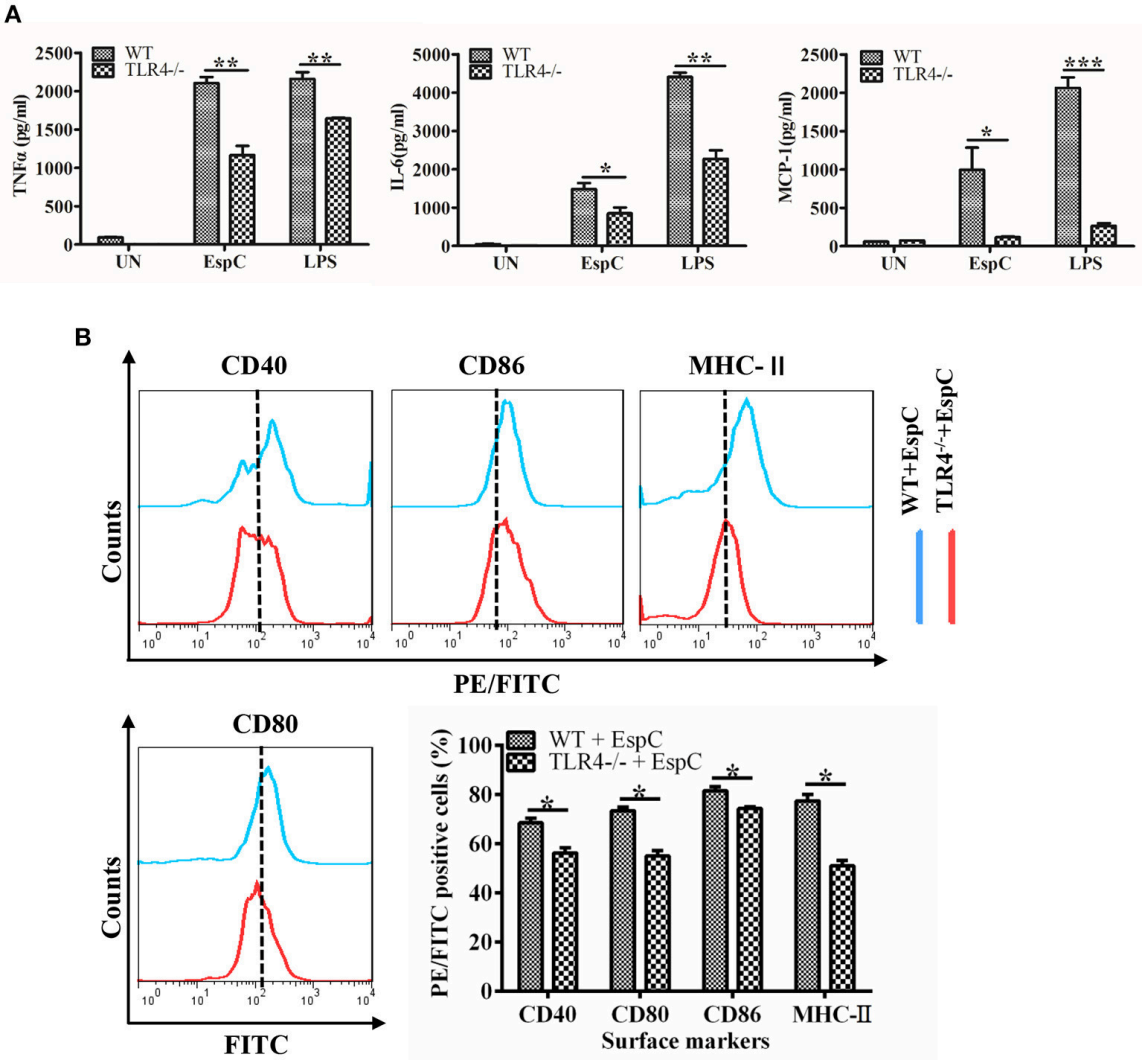
EspC enhanced the expression of pro-inflammatory cytokines and costimulatory and MHC molecules on macrophages in a TLR4-dependent manner. **(A)** TNF-α, IL-6, and MCP-1 production in macrophages derived from TLR4^−/−^ mice or C57BL/6 mice after treatment with medium alone, EspC (5 μg/mL), or LPS (1 μg/mL). Cytokine levels were measured by ELISA. **(B)** Macrophages from C57BL/6 mice and TLR4^−/−^ mice were treated with EspC (5 μg/mL) for 24 h and were analyzed to determine the expression of surface markers by flow cytometry. FACS analysis was repeated three times, and all samples were run in duplicate. Data are shown as means ± SEMs (*n* = 3); ^*^p < 0.05, ^**^p < 0.01, ^***^p < 0.001.

To conclusively demonstrate that the EspC-mediated expression of these pro-inflammatory cytokines was dependent on TLR4, we analyzed the responses of peritoneal macrophages from wild-type and TLR4^−/−^ mice to TLR2 and TLR4 ligands in the presence or absence of EspC. LPS was used as a positive control because it also binds to TLR4. EspC-dependent production of TNF-α, IL-6, and MCP-1 was greatly reduced in TLR4^−/−^ macrophages compared with that in wild-type macrophages (Figure 3A). Additionally, expression levels of CD40, CD80, CD86, and MHC-II were significantly decreased in macrophages from TLR4^−/−^ mice (Figure 3B). Taken together, these results sufficiently demonstrated that EspC was a ligand of TLR4, which is essential for EspC-induced activation of macrophages.

### EspC Induced the Phosphorylation of MAPKs

**Figure 4:**
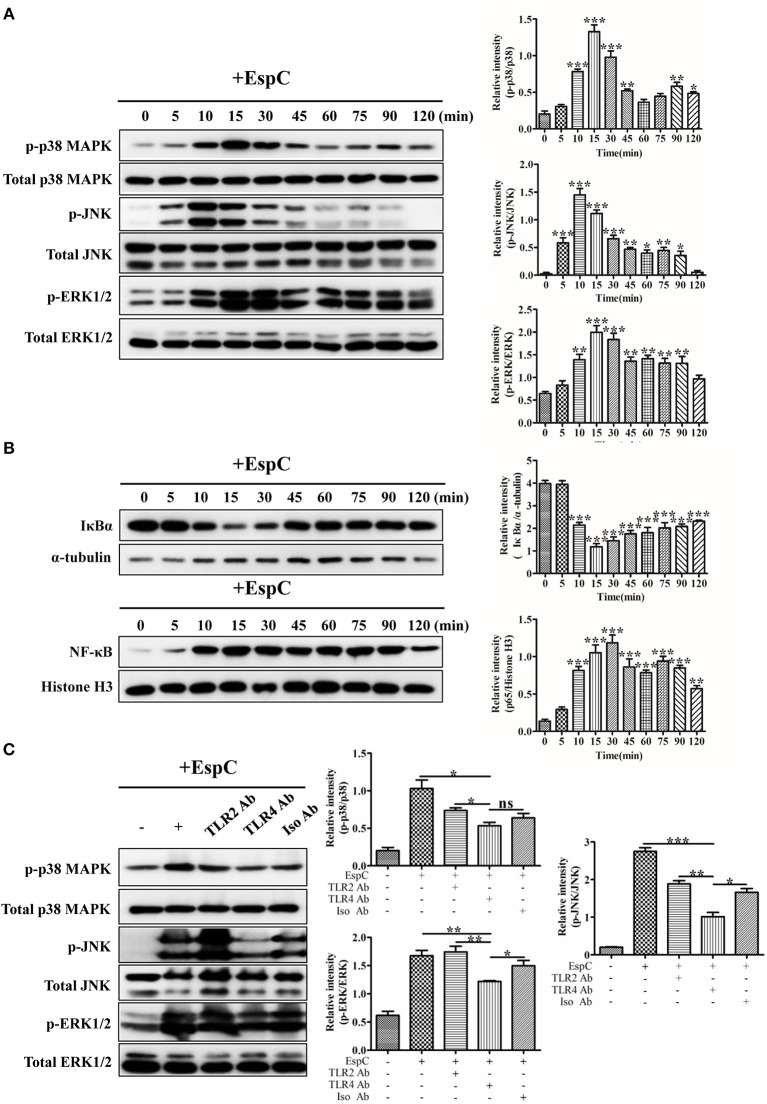
EspC induced the activation of MAPK and NF-κB. **(A)** RAW264.7 cells were incubated with EspC (5 μg/mL) for the indicated times (0, 5, 10, 15, 30, 45, 60, 75, 90, and 120 min). Cell lysates were then prepared, and the phosphorylation and activation of p38 MAPK, JNK, and ERK1/2 were examined by western blotting using specific antibodies targeting phospho-p38 MAPK (p-p38 MAPK), p38 MAPK, phospho-JNK (p-JNK), JNK, phospho-ERK1/2 (p-ERK1/2), and ERK1/2. **(B)** After incubation with EspC (5 μg/mL) for the indicated times, RAW264.7 cells were subjected to a protocol to separate cytoplasmic and nuclear fractions. The effects of EspC on the translocation of NF-κB from the cytoplasm to the nucleus and total IκBα levels in cytoplasmic extracts were determined by western blot analysis. **(C)** RAW264.7 cells were pretreated with blocking antibodies before stimulation with EspC (5 μg/mL). Western blotting analysis was performed to analyze the levels of p-p38 MAPK, p-JNK, p-ERK1/2, p38 MAPK, JNK, and ERK1/2. The relative band intensity of each protein was analyzed by Image J. Data are representative of three independent experiments and are shown as means ± SEMs (*n* = 3); ^*^p < 0.05, ^**^p < 0.01, ^***^p < 0.001.

The MAPK and NF-κB pathways are involved in the production of pro-inflammatory cytokines and activation of the host innate immune system (Li et al., 2015). To gain further insights into the mechanisms of EspC-derived macrophage activation, MAPK (p38 MAPK, JNK, and ERK1/2) phosphorylation, IκBα degradation, and NF-κB p65 nuclear translocation in macrophages were detected after stimulation with EspC using western blot analysis. As shown in Figure 4A, p38 MAPK, JNK, and ERK1/2 phosphorylation was enhanced after EspC stimulation (p38 MAPK and JNK: 10–30 min, ERK1/2: 10–90 min), peaking at 15, 10, and 15 min for p38 MAPK, JNK, and ERK1/2, respectively. In comparison, lower phosphorylation was observed in untreated cells. We also observed increased degradation of IκBα and nuclear translocation of NF-κB p65 during EspC stimulation (Figure 4B). Therefore, EspC-induced macrophage activation may be mediated by both the MAPK and NF-κB pathways.

To evaluate whether EspC-induced MAPK activation was dependent on TLR4, western blot analyses were performed to examine p38 MAPK, JNK, and ERK1/2 phosphorylation induced by EspC after pretreatment with anti-TLR2, anti-TLR4, or anti-IgG antibodies. As demonstrated in Figure 4C, JNK phosphorylation was strongly decreased, and p38 MAPK and ERK1/2 phosphorylation was also decreased when blocked with anti-TLR4 antibodies before EspC stimulation. In contrast, there were no significant differences in EspC-mediated MAPK phosphorylation between pretreatment with anti-TLR2 and anti-IgG control antibodies. Taken together, these results implied that TLR4 mainly mediated EspC-stimulated phosphorylation of MAPKs.

### Inhibition of the MAPK Pathway Prevented EspC-stimulated Expression of Cytokines and Surface Markers

**Figure 5:**
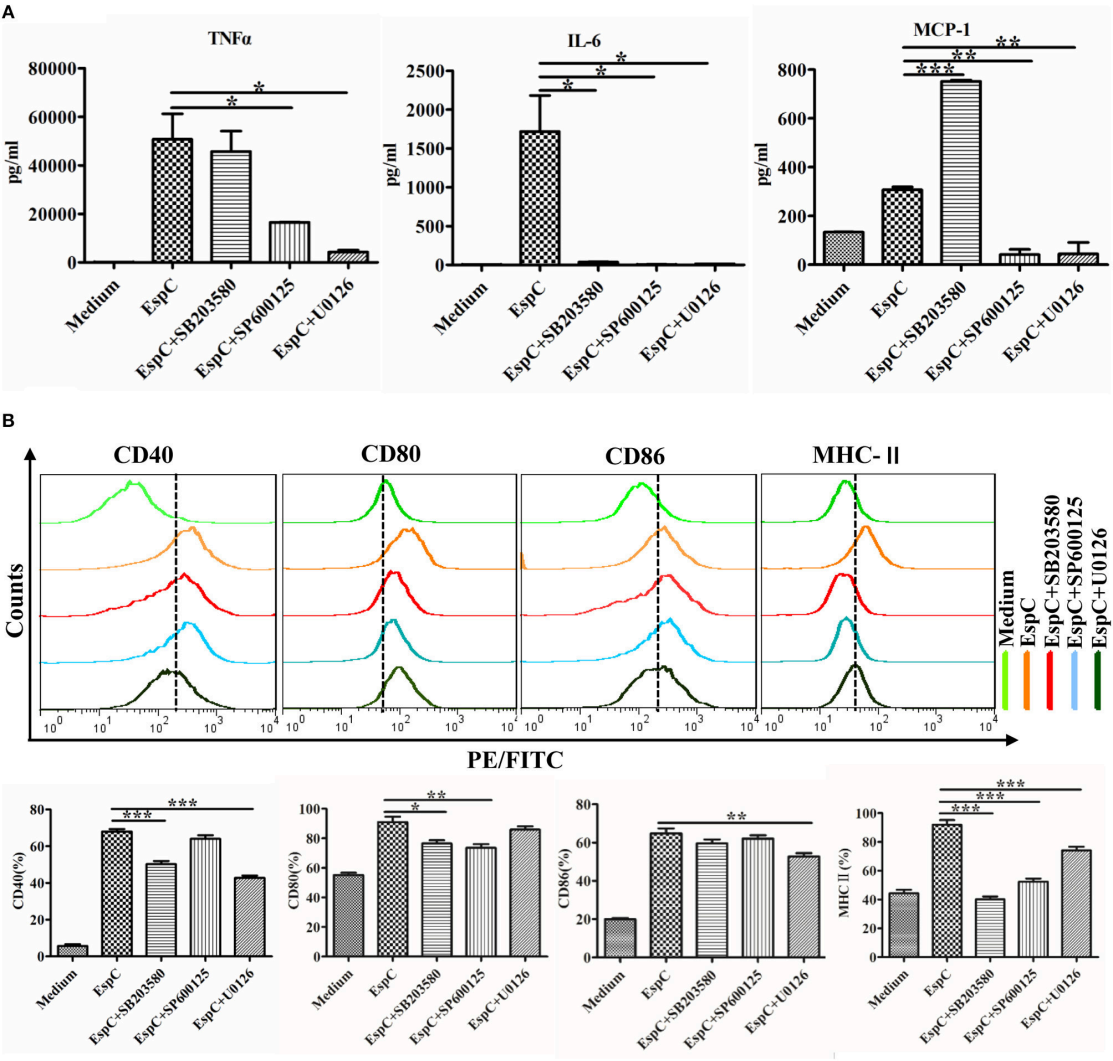
Involvement of the p38, JNK, and ERK pathways in EspC protein-induced production of pro-inflammatory cytokines and expression of costimulatory and MHC molecules. RAW264.7 cells were pretreated with pharmacological inhibitors of p38 (SB203580, 10 μM), JNK (SP600125, 25 μM), or ERK (U0126, 10 μM) or with dimethyl sulfoxide (vehicle control) for 1 h before being stimulated with 5 μg/mL EspC for 24 h. **(A)** Amounts of TNF-α, IL-6, and MCP-1 present in the culture medium were measured by ELISA. **(B)** The expression of CD40, CD80, CD86, and MHC-II was analyzed by flow cytometry. Values indicate means ± SEMs from three independent experiments; ^*^p < 0.05, ^**^p < 0.01, ^***^p < 0.001.

To confirm the effects of MAPKs on the EspC-induced expression of pro-inflammatory cytokines and surface markers, we pretreated RAW264.7 cells with or without highly specific kinase inhibitors (SB203580, a p38 MAPK inhibitor; SP600125, a JNK inhibitor; and U0126, an ERK1/2 inhibitor) followed by stimulation with EspC. As demonstrated in Figure 5A, the expression of IL-6 was significantly reduced by p38 MAPK, JNK, and ERK inhibitors, and the secretion of TNF-α and MCP-1 was inhibited by JNK and ERK inhibitors. Moreover, the p38 MAPK inhibitor decreased the expression of CD40, CD80, and MHC-II; the ERK1/2 inhibitor reduced the production of CD40, CD86, and MHC-II to varying degrees; and the JNK inhibitor only reduced the expression of CD80 and MHC-II (Figure 5B). Therefore, these results implied that JNK- and ERK1/2-dependent signaling pathways played major roles in the production of pro-inflammatory cytokines and that the expression of costimulatory molecules and MHC-II occurred mainly through the p38 MAPK and ERK1/2 signaling pathways.

### EspC Increased the Survival of *M. smegmatis* Within Macrophages

**Figure 6:**
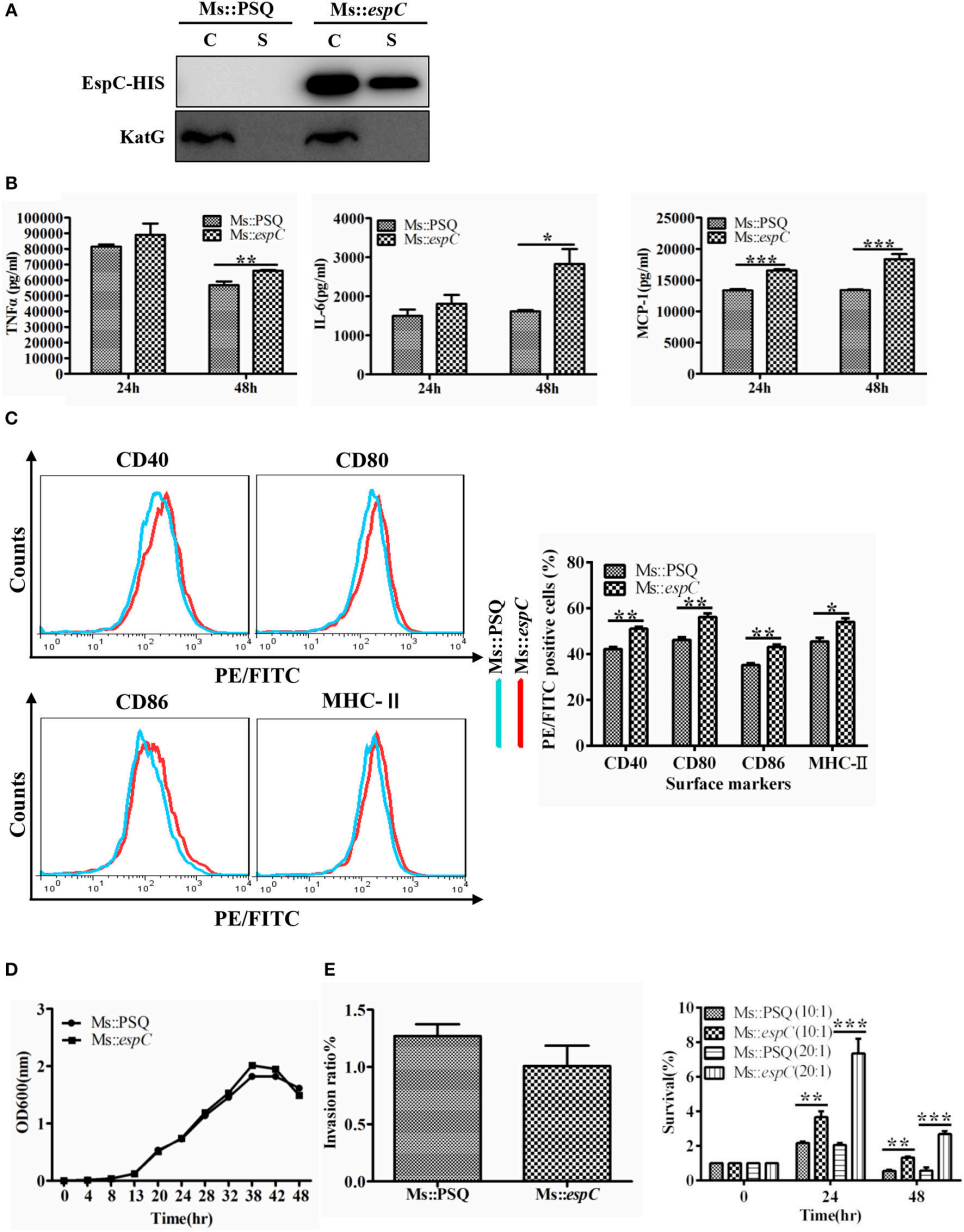
EspC increased survival of *M. smegmatis* within macrophages. RAW264.7 cells were infected for 24 or 48 h at an MOI of 10 with the indicated mycobacterial strains. **(A)** The two strains were cultured in Sauton containing 50 μg/mL kanamycin for 3 days, and both the bacteria and cell culture supernatant were then harvested for western blot analysis using mouse anti-His monoclonal antibodies. C: supernatants of bacterial supersonic lysates; S: cell culture filtrates. **(B)** Amounts of TNF-α, IL-6, and MCP-1 present in the culture medium were measured by ELISA. **(C)** The expression of CD40, CD80, CD86, and MHC-II was analyzed by flow cytometry. **(D)** Bacterial growth of Ms::*espC* and Ms::PSQ at 37°C in 7H9 broth supplemented with 10% OADC, as plotted using OD_600_ values determined every 6 h. **(E)** RAW264.7 cells were infected with Ms::*espC* or Ms::PSQ on day 0 (4 h after infection). We used an MOI of 10 for infection. Invasion ratios denote the ratio of the number of intracellular bacteria and the number of initial bacteria; RAW264.7 cells were infected with Ms::*espC* and Ms::PSQ at an MOI of 10 or 20. The infected macrophages were lysed at various time points after infection. Lysates containing live bacteria were diluted gradually and then plated on 7H10 agar plates to count bacterial cell numbers. The percent survival denotes the ratio of the number of intracellular bacteria at 24 and 48 h after infection and the number of intracellular bacteria at 0 h after infection. All results were generated from three independent experiments, and data are shown as means ± SEMs (*n* = 3); ^*^p < 0.05, ^**^p < 0.01, ^***^p < 0.001.

To further analyze the effects of EspC on macrophages in the context of the bacterium as a whole, we introduced *espC* from *M. tuberculosis* into *M. smegmatis* to obtain Ms::*espC*; transformants bearing the empty PSQ vector (Ms::PSQ), which failed to produce EspC, were used as a control. Western blot analysis showed that Ms::*espC* expressed His-tagged EspC protein in both the cell lysates and the cell filtrates, whereas Ms::PSQ did not (Figure 6A). The relative abilities of Ms::*espC* and Ms::PSQ to induce pro-inflammatory cytokines and surface markers in macrophages were evaluated. Consistent with EspC protein-induced pro-inflammatory cytokine production and surface marker expression, overexpression of EspC in *M. smegmatis* upregulated the expression levels of TNF-α, IL-6, and MCP-1 and promoted the expression of surface markers in macrophages compared with Ms::PSQ (Figures 6B,C).

To examine whether EspC from pathogenic mycobacteria affected the growth and survival of bacteria in the host cells, we compared growth properties between Ms::*espC* and Ms::PSQ under normal and intracellular conditions. The growth rate results showed that the growth of the two strains did not differ significantly under normal culture conditions (7H9 supplemented with 10% OADC and 0.05% Tween80; Figure 6D). However, by infecting macrophages with Ms::*espC* and Ms::PSQ at a similar invasion ratio, Ms::*espC* showed a higher survival rate in macrophages at 24–48 h after infection at different MOIs compared with *M. smegmatis* without EspC expression (Figure 6E). Taken together, these results indicated that EspC may contribute to the intracellular survival of mycobacteria within macrophages.

## Discussion

Mycobacterial components interact with host macrophages and modulate macrophage function to influence innate and adaptive immunities by inducing but not limit to production of either pro or anti-inflammatory cytokines to trigger a crosstalk between host and pathogen. We found that EspC greatly stimulated the generation of pro-inflammatory cytokines such as TNF-α, IL-6, and MCP-1 in RAW264.7 cells to promote the activation of macrophages. A recent study showed that EspC as an immunodiagnosis antigen also induced cytokine production of TNF-α, IL-6, and IFN-γ of pleural fluid mononuclear cells (PFMCs) from patients with tuberculous pleuritis (TBP) (Li et al., 2017). TNF-α is critical for granuloma formation and macrophage activation (Laster et al., 1988; Roach et al., 2002). IL-6 has pro-inflammatory activity, promotes the activation of macrophages, refers to induction of IFN-γ secretion, and initiates the protective Th1 immune response against *M. tuberculosis* (Flesch and Kaufmann, 1990; Ladel et al., 1997; Saunders et al., 2000). MCP-1 also plays a critical role in the host antimycobacterial inflammatory response and granuloma formation (Saunders and Britton, 2007; Majumder et al., 2008). Thus, these studies have suggested that EspC activates macrophages to facilitate the production of pro-inflammatory cytokines.

EspC significantly increased the expression of CD80, CD86, CD40, and MHC-II in RAW264.7 cells. T cells require two signals to become fully activated, and the co-stimulatory signal is necessary for T-cell activation. CD80 expressed on antigen-presenting cells (APC) interactions with CD28 results in T-cell activation and differentiation, whereas CD86 binding to cytotoxic T-lymphocyte antigen-4 suppresses T-cell receptor signaling (Sharpe, 2009). Therefore, EspC-induced increase in CD80 and CD86 may function synergistically to confer protective immune responses and promote T-cell activation. MHC class II molecules bound to antigen peptides can be recognized by CD4^+^ T cells, which is vital for enhancing protective T-cell responses. Moreover, ligation of CD40 to macrophages can stimulate IL-12 production (Lazarevic et al., 2003), which can prime IFN-γ-producing T cells crucial for the control of *M. tuberculosis* infection. EspC is actively expressed and accessible in the antigen-processing process during intracellular *M. tuberculosis* infection *in vivo* (Fisher et al., 2002; Rodriguez et al., 2002). The results of this study indicate that EspC can promote the macrophage functions of antigen presentation and lymphocyte activation and can explain why EspC may be a good tuberculosis vaccine candidate and T-cell-based immunodiagnosis antigen.

Generally, TLRs on APCs initiate innate immune responses and modulate adaptive immune responses through the recognition of microbial molecules. The pathogenesis and virulence of *M. tuberculosis* also depend on TLRs and their signaling cascades. It has been shown that ESAT-6 (EsxA) binds directly to TLR2, activates Akt, and abrogates NF-κB activation to lower innate immune responses and favor intracellular pathogen cell-to-cell spreading (Pathak et al., 2007). Unlike the role of ESAT-6 in decreasing the production of pro-inflammatory cytokines and innate immune responses, we found that EspC induced macrophage activation by directly binding to TLR4 and triggering intracellular signaling of MAPK pathways, and nuclear NF-κB p65 was greatly enhanced in EspC-treated macrophages. MAPK and NF-κB are two major immune signaling pathways that mediate the regulation of innate immune responses (Li et al., 2015). In addition, *M. smegmatis* overexpressing EspC induced proinflammatory cytokine increase and enhanced the expression of surface markers. These results indicate that EspC is a factor from the ESX-1 system and greatly affects macrophage activation and antigen presentation via TLR4/MAPK pathways to activate the innate immune response.

Presently, there are no considerable data on the function and pathogenicity of EspC protein. A recent study showed that *M. tuberculosis* EspC forms a filamentous structure in the cell envelope of *M. tuberculosis* that may act as an ESX-1 secretion channel (Lou et al., 2017). The C-terminal amino acid residues (98–103) of EspC contribute to filamentous structure formation, protein stability, and export (Lou et al., 2017). In our study, we found that EspC can improve resistance to oxidative stress and increase the survival of *M. smegmatis* within macrophages, suggesting that EspC may be an important virulence factor of pathogenic mycobacteria and might be beneficial for mycobacteria cell-to-cell spread within the host. The *espACD* operon has been suggested to be a pathogenicity-associated genome island (Simeone et al., 2012; Majlessi et al., 2015) owing to its distribution in the genomes of certain slow-growing, opportunistic or obligate mycobacterial pathogens. The presence of the *espACD* operon in pathogenic mycobacteria has been assumed the result of the independent horizontal gene transfer events, which contribute to pathogenicity (Gröschel et al., 2016). *M. tuberculosis* is able to escape from phagosomes into macrophage cytosol; this is dependent on EAST-6 secretion by the ESX-1 system (de Jonge et al., 2007), and loss or gain of mycobacterial virulence is also ascribed to ESAT-6 secretion by mycobacteria. In fact, similar to BCG, deletion of the *Esx-1* locus, known as RD1 in *M. tuberculosis* and *M. bovis* strains, also blocked the secretion of EspC; this is because EspC secretion and EAST-6 secretion are mutually dependent. Given that EspC can mediate the activation of macrophages and increase the survival of mycobacteria within macrophages, it is impossible to deny the role of EspC in the interaction between pathogens and macrophages. Additionally, these findings imply that the virulence and interactions between macrophages and ESX-1 proteins are not strictly correlated with EsxA.

Many important questions remain unanswered. In this study, we established that EspC was an antigen that greatly affected macrophage activation and antigen presentation by directly binding to TLR4 and triggering intracellular MAPK signaling pathways to activate the innate immune response. However, further studies are needed to reveal the underlying mechanisms mediating the interactions between EspC and macrophages or innate immune responses. EspC can increase survival of *M. smegmatis* within macrophages. *M. smegmatis* also has an endogenous ESX-1 system, which can secrete ESAT-6 from *M. smegmatis* (MsESAT-6), although MsESAT-6 is not able to rupture phagosomal membranes (De Leon et al., 2012). A previous study reported that *M. tuberculosis* ESAT-6 was secreted by the endogenous ESX-1 system of *M. smegmatis* (Converse and Cox, 2005). We hypothesized that Mtb EspC may also be secreted by the endogenous ESX-1 system in *M. smegmatis*. Although there is no homolog for Mtb EspC in *M. smegmatis*, as a cosecreted factor by the ESX-1 system in *M. tuberculosis*, whether Mtb EspC expression may affect the function of the ESX-1 system of *M. smegmatis*, thereby inducing a stress response that indirectly protects survival of the Ms*::espC* strain, is still unclear and should be evaluated in future studies. Considering the key roles of the secretion system in immunity and the pathogenesis of bacterial infection, it would be interesting to gain deeper insights into the exact roles of ESX substrates in macrophage-mycobacteria interactions, which may contribute to a better understanding of complex host-pathogen interactions and affect the design of new tuberculosis vaccines with enhanced immunogenicity.

## Ethics Statement

This study was carried out in accordance with the recommendations of the Guide for the Care and Use of Laboratory Animals from the National Institutes of Health. The experimental protocol was approved by the Animal Care and Use Committee of Fudan University.

## Author Contributions

QG performed the experiments. JB helped to perform EspC secretion assay needed in our revised manuscript. ML helped to construct the vector expressing EspC. XZ, QG, WG, YX, and WF analyzed the data. XZ and QG designed the study and wrote the paper. HW and XZ reviewed the paper and supervised the research. All authors have revised the paper critically and approved the submitted and final versions.

### Conflict of Interest Statement

The authors declare that the research was conducted in the absence of any commercial or financial relationships that could be construed as a potential conflict of interest.
